# Proposing Causal Sequence of Death by Neural Machine Translation in Public Health Informatics

**DOI:** 10.1109/JBHI.2022.3163013

**Published:** 2022-04-14

**Authors:** Yuanda Zhu, Ying Sha, Hang Wu, Mai Li, Ryan A. Hoffman, May D. Wang

**Affiliations:** School of Electrical and Computer Engineering, Georgia Institute of Technology, Atlanta, GA 30332 USA; School of Biology, Georgia Institute of Technology, Atlanta, GA 30332 USA; Department of Biomedical Engineering, Georgia Institute of Technology and Emory University, Atlanta, GA 30332 USA; Department of Electronic Science and Technology, University of Science and Technology of China, Hefei 230026, China; Department of Biomedical Engineering, Georgia Institute of Technology and Emory University, Atlanta, GA 30332 USA; Department of Biomedical Engineering and School of Electrical and Computer Engineering, Georgia Institute of Technology and Emory University, Atlanta, GA 30332 USA

**Keywords:** Cause of death, COVID-19 pandemic, deep learning, fast healthcare interoperability resources (FHIR), population health data analytics

## Abstract

Each year there are nearly 57 million deaths worldwide, with over 2.7 million in the United States. Timely, accurate and complete death reporting is critical for public health, especially during the COVID-19 pandemic, as institutions and government agencies rely on death reports to formulate responses to communicable diseases. Unfortunately, determining the causes of death is challenging even for experienced physicians. The novel coronavirus and its variants may further complicate the task, as physicians and experts are still investigating COVID-related complications. To assist physicians in accurately reporting causes of death, an advanced Artificial Intelligence (AI) approach is presented to determine a chronically ordered sequence of conditions that lead to death (named as the causal sequence of death), based on decedent’s last hospital discharge record. The key design is to learn the causal relationship among clinical codes and to identify death-related conditions. There exist three challenges: different clinical coding systems, medical domain knowledge constraint, and data interoperability. First, we apply neural machine translation models with various attention mechanisms to generate sequences of causes of death. We use the BLEU (BiLingual Evaluation Understudy) score with three accuracy metrics to evaluate the quality of generated sequences. Second, we incorporate expert-verified medical domain knowledge as constraints when generating the causal sequences of death. Lastly, we develop a Fast Healthcare Interoperability Resources (FHIR) interface that demonstrates the usability of this work in clinical practice. Our results match the state-of-art reporting and can assist physicians and experts in public health crisis such as the COVID-19 pandemic.

## I. Introduction

There are more than 2.7 million deaths in the United States [1] and nearly 57 million deaths around the world per year.^1^ As of March 23rd, 2022, coronavirus has taken the lives of nearly 6.1 million people among 472 million confirmed cases globally.^2^ Even though COVID-19 is ranked as the third leading cause of death [2][3], detailed information on COVID-19 related complications and causes of death are still under investigation [4]–[7]. Therefore, accurate death reporting is essential for public health institutions such as the U.S. National Center for Health Statistics (NCHS) and the Centers for Disease Control and Prevention (CDC) to formulate effective recommendations.

The U.S. death reporting system requires two types of causes of death to be filled on death certificates: a *single* medical condition that is the underlying cause of death, and an ordered sequence of medical conditions (a sequence of ordered causes, which is termed “causal sequence” in our context) that lead to the death. These sequences of causes of death form the basis of the NCHS Multiple Causes of Death data, which is a critically valuable data source in public health.

A causal sequence of death consists of one underlying cause of death, and other potential immediate causes of death. The immediate causes of death are typically caused by the underlying cause of death. An example causal sequence of death is “chronic obstructive pulmonary disease, unspecified (ICD10: J44.9) → other disorders of lung (ICD-10: J98.4)”. Here ICD-10 stands for “10th revision of the International Statistical Classification of Diseases and Related Health Problems,” a common coding system used in death reporting.^3^

The process of determining causal sequences of death is challenging, even for experienced physicians, as this process involves careful reasoning using medical domain knowledge and experience. In addition, limited electronic health records in cases of sudden death may significantly complicate the determination of correct sequences.

Complete and accurate reporting of condition sequence leading to death provides an invaluable public health resource for tracking disease prevalence, developing public health interventions, and tracking intervention efficacy over time. Thus, it improves both clinical care and patient well-being. For physicians and public health experts, frequently reported sequences can assist in grouping disease conditions, and discovering underlying causal relationships that have not been previously observed. At the patient level, such sequences can alert individual patients for early actions before symptoms shown.

To assist in timely, accurate, and complete reporting of deaths and to reduce the subjectivity by reporting physicians, we develop a decision support system with deep learning approaches that learns the causal relationship between death and available clinical codes, and generates the causal sequence of death based on the decedent’s disease histories. Table I summarizes three challenges and the proposed solutions.

The first challenge is due to the use of different coding systems of clinical conditions. The existing causes of death in the U.S. have been using the tenth revision (ICD-10) codes since January 1999 [8]. On the other hand, healthcare institutions and practitioners in the U.S. were still filing patients’ health record using the ninth revision (ICD-9) codes until October 2015 [9]. ICD-10 codes are quite different from ICD-9 codes in both coding structure and quantity: ICD-10 has nearly five times as many diagnosis codes as ICD-9.^4^

One solution to this challenge is natural language translation. The input sequence to our model is diagnosis codes from the last hospital discharge record of the deceased, and the output sequence is the corresponding causes of death for that decedent. Similar to translating English sentences to French sentences, we propose a succinct causal sequence of death in ICD-10 codes from the priority-based discharge records of ICD-9 codes. The area of Natural Language Processing (NLP) contains extensive studies for machine translation, such as autoregressive [10]–[13] and autoencoder models [14]–[16]. The former factorizes the probability of a given corpus into a series of conditional probabilities while the latter generates output through reconstructing corrupted input.

The second challenge is the domain knowledge requirement. As a data-driven approach, a deep learning model can sometimes generate confusing sequences to the physicians or results contradicting medical domain knowledge. Consequently, the physicians may find it difficult to trust the generated results. To solve this problem, we incorporate medical domain knowledge in the deep learning framework. Particularly, we use an external source of expert-curated rules, which are pairs of causal relationships between clinical condition codes. When the deep learning model searches for the next clinical condition in generating the output sequence, only clinical conditions following medical domain knowledge can serve as candidates.

The last challenge is the data interoperability in death reporting. Currently, the U.S. National Center for Health Statistics coordinates with 57 reporting jurisdictions across the United States to aggregate mortality data [17]. These reporting jurisdictions have different regulations and local laws. To streamline the data storage and transmission between hospitals and these public health institutions and to make data comprehensive for future Big Data analytics, we use Fast Healthcare Interoperability Resources (FHIR) [18] to standardize mortality data reporting. We have developed one web-based FHIR application [19] to access electronic health records data. The newly developed Android version mobile application is FHIR compatible; it can pre-populate different sections of death certificate to extract essential information of health history of the decedents. Furthermore, it serves as a graphical user interface for physicians that the mobile application can automatically query the deep learning models to provide clinical decision support. Implementation details, graphic user interface and video demo information are included in the supplementary file.

**Fig. 1.**
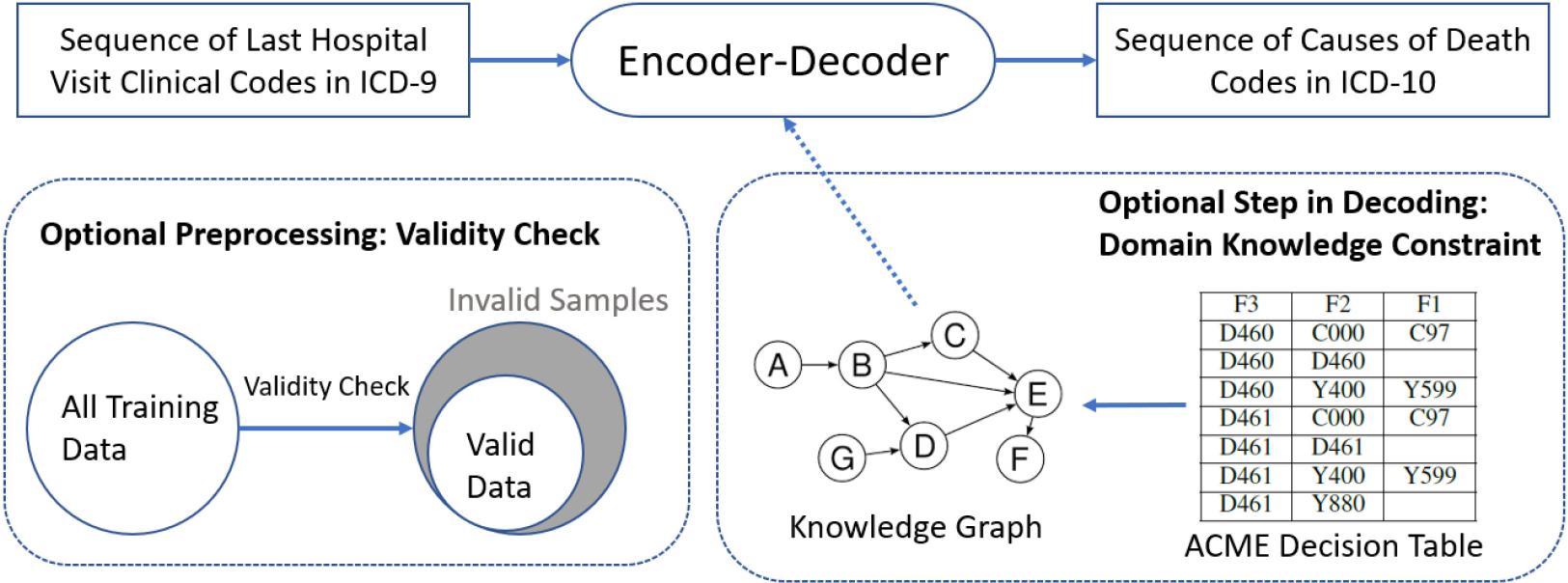
Overall Structure of this paper. The encoder-decoder model is the main framework for generating sequences of causes of death. Validity check is an optional preprocessing step and domain knowledge constraint is an optional step in decoding.

In this work, we predict the sequence of causes of death from decedent’s last hospital discharge record using encoder-decoder models with attention mechanism. We also visualize the attention scores to identify death-related conditions from unrelated symptoms. We further demonstrate the feasibility of the encoder-decoder models for ICD-10 input data by mapping ICD-9 codes to ICD-10 codes to meet current electronic health records (EHRs) data. In addition, we learn the expert domain knowledge graph from an ACME (Automatic Classification of Medical Entry) decision table to constrain model predictions to known relationships. The overall structure is shown in Fig. 1.

In summary, this work has the following contributions:

1. This is the first work to develop encoder-decoder models for predicting causal sequences of death based on death reports and decedents’ last hospital visit records;
2. This is the first work to identify death-related conditions from available health records using attention visualization. Our approach improves model interpretation and can potentially benefit physicians in predicting causes of death;
3. This is the first work to use the modified BLEU (BiLingual Evaluation Understudy) score, a popular score for sequence-to-sequence translation task in natural language processing, to evaluate the performance of deep learning prediction of causal sequence of death;
4. This work improves data interoperability by implementing a user-friendly, FHIR-based application to utilize AI solutions.

## II. Recent Work

Intelligent death reporting has been a rising research theme in recent years. Jiang *et al.* [20] applied topic modeling on the multiple causes-of-death U.S. mortality data from NCHS between 1999 and 2014. The authors successfully grouped comorbidities based on their correlation and explore the temporal evolution of these morbidity groups. Unfortunately, due to the nature of unsupervised learning, the author failed to determine the optimal number of topic groups, reducing its potential impact on clinical practice. Wu and Wang [21] designed a convolutional neural network (CNN) with dynamic computation graph to infer the underlying cause of death using the same NCHS mortality data. Using a list of relevant medical conditions, the proposed CNN model was able to achieve 75% accuracy in predicting the single underlying cause of death. Meanwhile, Hoffman *et al.* [22] revealed the poor quality of death reporting data by showing 20.1% discordance of cause of death. The author also proposed validity checking on death reporting data to remove invalid causal pairs of death codes. One limitation is that, the author did not validate any downstream tasks, such as predicting the single underlying cause of death, to further demonstrate the value of validity checking.

A recent yet interesting work published on Journal of Biomedical and Health Informatics [23] is to automatically extract the single cause of death from verbal autopsy questionnaire using recurrent neural network (RNN) with attention. The RNN model with attention is able to learn the textual representation from the free-text questionnaire data and visualize attention scores to improve outcome interpretation. RNN models are also applied to mortality prediction. Yu *et al.* [24] proposed a multi-task RNN model with attention mechanisms that predicts patients’ hospital mortality and achieved higher sensitivity than the simplified acute physiology score (SAPS-II). The auxiliary task in the proposed multi-task RNN model is the reconstruction of patients’ physiological time series data.

## III. Causal Sequence of Death

### A. Data

**Fig. 2.**
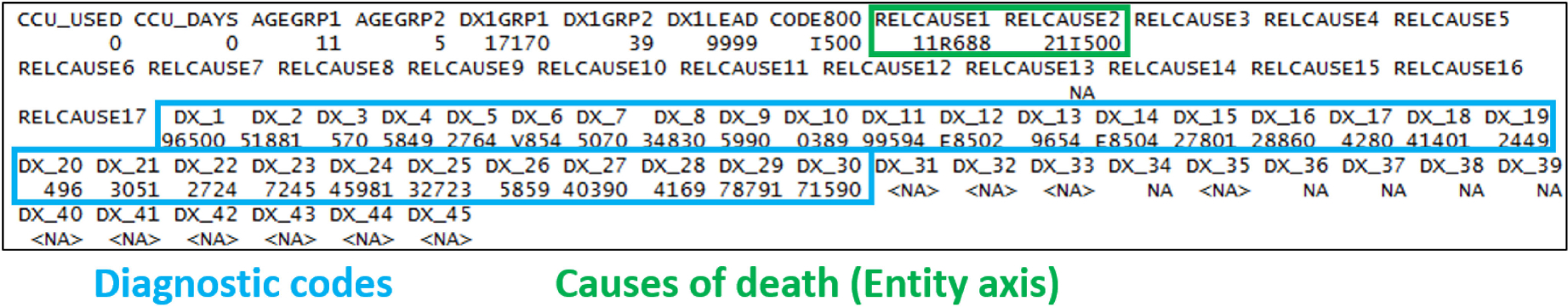
Sample data from the Michigan data set. The casual sequence of death in ICD-10 for this decedent is *I*500 > *R*688 (Heart failure > Other general symptoms and signs), outlined in green. This decedent had a total of 30 ICD-9 diagnostic codes assigned during the last visit to hospital, outlined in blue.

In this work, we use last hospital visit discharge records from Michigan Vital Statistics Data that covers 181,137 decedents. This dataset was collected by CDC and its collaborators before 2017 and contains important demographic information, diagnostic codes and procedural codes. However, this dataset does not include decedents’ past medical histories (no temporal information; last hospital visits only). As shown in Fig. 2, each decedent has exactly one line of last hospital visit essential information, including up to 45 clinical diagnosis codes, one underlying cause of death and up to 17 related causes of death. On average, each decedent has 18.84 diagnosis codes and 2.25 causes of death (including the underlying cause of death). In line with the ICD-9-CM Official Guidelines for Coding and Reporting,^5^ the diagnosis codes are in priority-based sequence of ICD-9 codes. The causes of death are in ICD-10 codes. Typically, we have a longer input source sequence around 16 to 20 codes, and a much shorter output target sequence with roughly two to three codes. Such a short sequence of death codes is expected in death reports. We accessed the ten years’ (2009 to 2018) NCHS Mortality Multiple Cause Files database^6^ and calculated that the average length of death code sequence among 26,322,220 decedent samples to be 2.95 codes. (Note that discharge codes on last hospital admission may contain previous admission discharge codes.)

ACME (Automatic Classification of Medical Entry) is an ontology of medically valid causal relationships between ICD-10 codes developed, improved, and promulgated by an international team of medical experts [25]. The ACME decision table was used to learn the medical domain knowledge constraint [22]. It contains 95,321 lines of causal relationship. Specifically, if rules are of length 2, it can be interpreted as F2 → F3 (cause of death code F2 leading to cause of death code F3); if rules are of length 3, it can be represented as (F1:F2) → F3 (all codes within the subset are cause of death that lead to cause of death code F3). The ACME decision table was transformed into a knowledge graph; nodes are diagnosis codes and directed edges were pairwise rules.

### B. Generating Causal Sequences Through Translation

We can define the generation of causal sequences as follows:

*Definition 1:* [Generation of Causal sequences] Given a deceased’s medical history represented as a collection of clinical codes **x** = *x*_1_,…,*x*_*m*_, the goal of causal sequence generation is to identify a list of clinical codes **y** = *y*_1_,…,*y*_*n*_ that orders the conditions leading to death.

The objective is to generate the causal sequence of death, an ordered sequence of causes of death codes in ICD-10. The input is a sequence of diagnosis codes in ICD-9. To generate the output sequence from one domain based on the input sequence from another domain, we apply the state-of-the-art algorithms from neural machine translation.

Input and output sequence data are split into training, validation and testing set at the ratio of 7:1:2. We applied five-fold cross validation. We achieved similar results using ten-fold cross validation (the split is 8:1:1). More results are in the supplementary file.

## IV. Methodology

### A. Neural Machine Translation: Encoder and Decoder

**Fig. 3.**
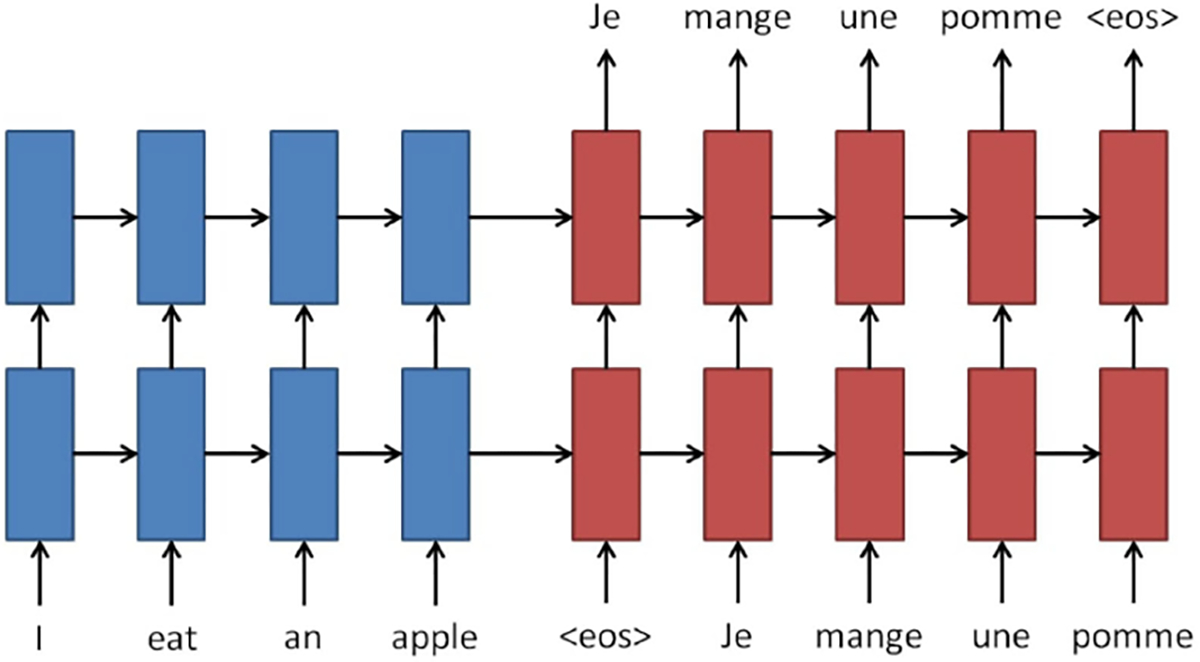
Neural machine translation consists of an encoder (stacked recurrent networks in blue) and a decoder (stacked recurrent networks in red). The symbol < *eos* > is a special token referring to the end of a sentence. Adapted from [13].

The goal of translation is to find a target sentence **y** = *y*_1_,…,*y*_*n*_ which maximizes the conditional probability *p*(**y**|**x**) given a source sentence **x** = *x*_1_,…,*x*_*m*_. Neural machine translation (NMT) aims to maximize this conditional probability of source-target sentence pairs by using a parallel training corpus to fit a parameterized model. As shown in Fig. 3, there are two basic components of an NMT system:

1. An encoder encodes the input sequence **x** into representation **s**
2. A decoder generates the output sequence **y**

The conditional probability of the decoder is formulated as:

(1) $$\text{log}\;p(\text{y}|\text{x})=\sum_{t=1}^{n}\text{log}p\left(y_{t}|y_{1},y_{2},\ldots ,y_{t-1},\text{s}\right)$$

The probability of the next generated word *y*_*i*_, is jointly decided by the learned representation vector **s** and all previously generated words *y*_1_,…,*y*_*t*−1_.

#### 1) LSTM Encoder - LSTM Decoder:

In an long short-term memory (LSTM) Encoder-Decoder framework [26], [27], the encoder reads and encodes an input sequence of embedded vectors **x**. The encoder will then generate a hidden state *h*_*t*_ at time *t* from the current input *x*_*t*_ and the previous hidden state *h*_*t*−1_:

(2) $$h_{t}=f\left(x_{t},h_{t-1}\right)$$

The source input representation vector **s** shall have the form:

(3) $$\text{s}=q\left(h_{1},\ldots ,h_{m}\right)$$

Here *f* and *q* are some non-linear functions. For the basic recurrent neural network RNN/LSTM model, the conditional probability of output sequence **y** at time *t* can be written as:

(4) $$p\left(y_{t}|y_{1},\ldots ,y_{t-1},\text{s}\right)=g\left(y_{t-1},h_{t},\text{s}\right)$$

Here *g* is a (multi-layered) nonlinear function.

Generic RNN or LSTM encoder-decoder framework has to process the sentence word by word using fixed length vectors, failing to preserve long-term dependency. Bahdanau *et al.* proposed soft alignment (soft attention) [10] in a bi-directional RNN model that enables the model to search for a (sub)set of input words or encoded representation vectors when generating each target word. The soft attention score is calculated as:

(5) $$score\left(s_{t},h_{i}\right)=v_{a}^{T}\text{tanh}\left(W_{a}s_{t-1}+U_{a}h_{i}\right)$$

Where *s*_*t*_ = *f*(*s*_*t*−1_, *yt* − 1, *c*_*t*_) is the hidden state of output word *y*_*t*_ at position *t*, the context vector *c*_*t*_ is the weighted sum of hidden states of the input sequence, and *W*_*a*_, *U*_*a*_, *v*_*a*_ are trainable matrices.

Luong *et al.* [12] proposed global attention which predicts the position of alignment for the current word before computing the context vector using the window centered around that source position. The general attention score, a sub-category of the global attention mechanism, is calculated as:

(6) $$score\left(s_{t},h_{i}\right)=s_{t}^{T}W_{a}h_{i}$$

Here *W*_*a*_ is a trainable weight matrix in the attention layer.

Global attention [12] and soft attention [10] are “similar in spirit,” but there is a major difference. Global attention uses hidden states from the top LSTM layers of both encoders and decoders, while soft attention uses the concatenation of forward and backward hidden states in the bi-directional RNN encoder.

Overall, the LSTM encoder-decoder model is easy to understand, and can be applied on most sequence-to-sequence tasks. Yet such a model has limited performance, especially on long sentences.

#### 2) Bidirectional RNN Encoder - LSTM Decoder:

A major disadvantage of the traditional encoder-decoder model is that the neural networks compress source sentences into fixed-length vectors. This may significantly limit the capability of translating long sentences [28]. Bahdanau proposed a bidirectional RNN [10] with soft alignment so that the model can learn to align and translate jointly. A bi-directional RNN encoder model can better learn the embedding of words, but it is less efficient than the LSTM encoder-decoder framework, and has less accurate results than transformer models.

#### 3) Transformer Model:

**Fig. 4.**
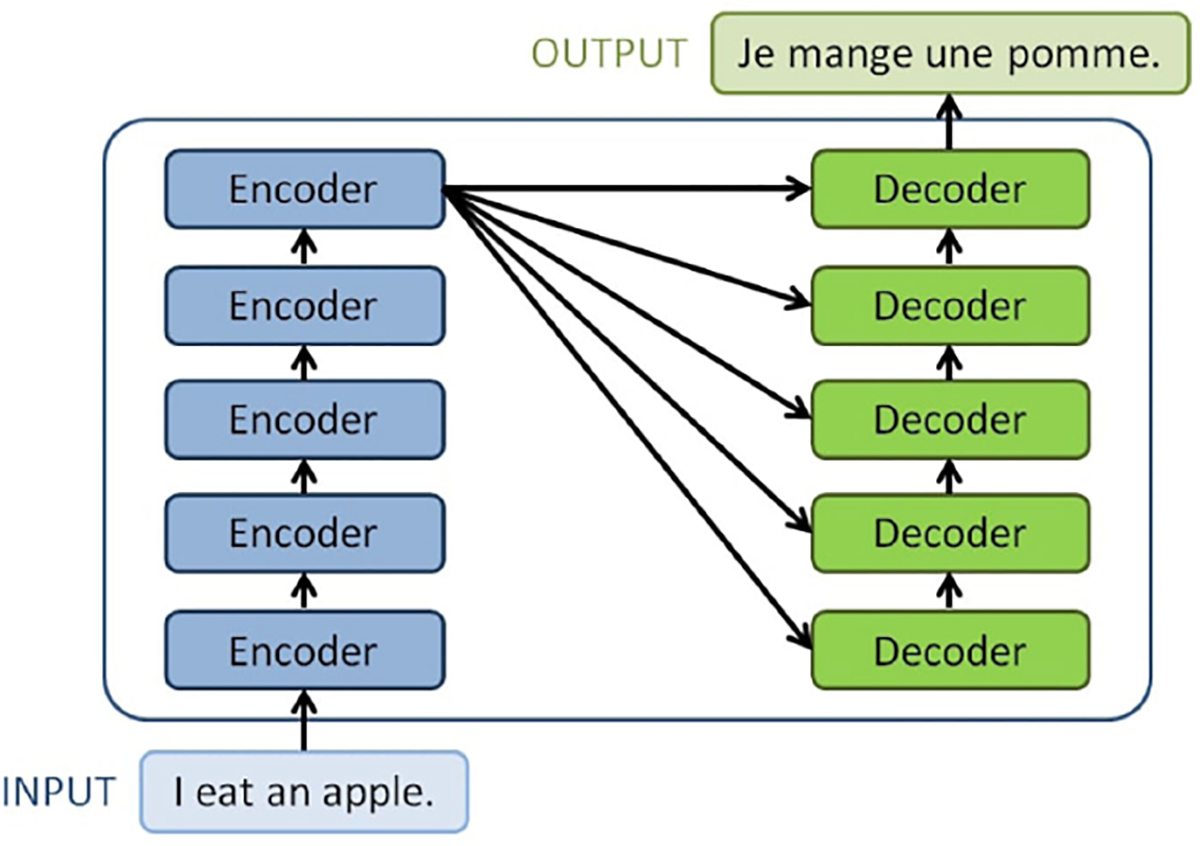
Overall structure of a transformer. Here we have five identical encoders and five identical decoders in this transformer.^7^

Still, RNN-based encoder-decoder models fail to perform well on long sentences. To overcome this problem, Vaswani *et al.* proposed the transformer framework with multi-head self attention module [29] that enables encoding words of the same sentence in parallel. As shown in Fig. 4, a transformer consists a stack of encoders and the same number of decoders. The embedded input is passed to the encoder at the bottom; the output from the encoder on the top will be passed to all decoders. The decoder on the top will pass the output to a linear layer and a softmax layer to generate a predicted sentence. The encoder has two layers: a multi-head self-attention layer and a feed forward layer. The decoder has an extra multi-head attention layer that processes both the output from the encoder stack and the output from previous attention layer.

The self attention module is the core component of the transformer model. The attention score is a scaled dot-product of matrices *Query, Key* matrices *Q, K*, or the weighted sum of the *Value* matrix *V*.


(7) $$Attention(Q,K,V)=softmax\left(\frac{QK^{T}}{\sqrt{d_{k}}}\right)V$$

The *Query, Key* and *Value* matrices are generated through linear transformation *Q* = *XW*^*Q*^, *K* = *XW*^*K*^, *V* = *XW*^*V*^, Where *W*^*Q*^, *W*^*K*^, *W*^*V*^ are learnable parameters.

The transformer model is more time-consuming to train than RNN-based encoder-decoder frameworks, but can achieve far better results [29]. BERT (Bidirectional Encoder Representations from Transformers) [14] is a transformer encoder model that has been pre-trained on large datasets (BooksCorpus with 800 M words and English Wikipedia with 2,500 M words). The pre-trained BERT model can be further fine-tuned to improve performance on multiple NLP tasks.

### B. Decoding and Translation

A straightforward method of decoding is to predict only one word with the highest score based on previous steps. It is efficient and easy to understand; yet a small mistaken output might corrupt all remaining predictions. Thus, a better strategy named “beam search” [30] is adopted. In each step of the decoding process, the decoder generates multiple candidates based on a previous output, and each of these candidate has a non-zero probability value. Beam search keeps the top *k* candidates for each step, keeps track of all paths of candidate outputs, and selects the path of highest overall probability when reaching the end of the sequence. Here, *k* is the beam size. The larger the value of *k* is, the more robust the decoding process is; yet this may require more memory and increase computational time.

We also include medical domain knowledge as constraints during translation. The ACME decision table specifies all the “feasible” pairwise causal relationships between ICD diagnosis codes [22][25]. Using this decision table, we construct a domain knowledge graph on all diagnosis codes from Michigan data before training. With diagnosis codes as nodes, we add directed paths between them only if such causal relationship can be found in the ACME decision table. When decoding, the networks are required to look up the knowledge graph and only include “feasible” codes in the top *k* hypotheses.

### C. Evaluation

For quantitative evaluation, we evaluate how well our proposed causal chain $\hat{Y}=\left\{{\hat{Y}}_{1},\ldots ,{\hat{Y}}_{M_{1}}\right\}$ aligns with the physicians’ decision, i.e., $Y=\left\{Y_{1},\ldots ,Y_{M_{2}}\right\}$. Here *Y*_*i*_ is the individual codes, and *M*_1_, *M*_2_ are the respective length of the chains. A perfect alignment means *M*_1_ = *M*_2_, and ${\hat{Y}}_{i}=Y_{i}$, for *i* = 1, …, *M*_1_. However, this is rarely the case, thus we compute a weighted average precision of our alignment in sub-sequences of variable lengths, i.e., the BLEU score [31]. Following natural language processing literature, we call sub-sequence of length *i* “i-grams”. BLEU score ranges from 0 to 1 or (or from 0 to 100 if multiplied by 100), and the higher BLEU, the higher we have an alignment with physicians clinically.

A simple example follows illustrates the computation of the BLEU score. In our proposed candidate sequence, the underlying cause of death, *Asphyxia and Hypoxemia (R909)* leads to *Pneumonia, Unspecified Organism (J189)* which leads to *Respiratory failure, unspecified (J969)*.


$$\hat{Y}=R909\to J189\to J969$$

The reference sequence, determined by the physician, consists of *Asphyxia and Hypoxemia (R909)*, *Pneumonia, Unspecified Organism (J189)* and then *Acute Respiratory Failure (J960)*.


Y=R909→J189→J960

As shown in Table II, we first list 1-grams and 2-grams from $\hat{Y}$ and *Y*, and we compute the precision for the two cases. Here the definition of precision is similar in the classification setting: among all the predictions we made in candidate sequence $\hat{Y}$, the number of candidate sequences we get correct in the reference sequence *Y*. After we compute all the precision metrics, we calculate the geometric average of them as the BLEU metrics, approximately 0.47.

In natural language settings, people usually calculate BLEU score for the geometric average up to 4-gram precision. In our case, however, we only compute the geometric average up to 2-gram precision, and apply clipping to each of the precision. This is due to the fact that the average length of causal chain of death in Michigan dataset is 2.25 codes so including 3-gram precision will lead to substantially inaccurate evaluation. Furthermore, we also include a brevity penalty to penalize sentences that are too short.

According to [31], the modified i-gram precision is defined as:

(8) $$p_{i}=\frac{\forall \;\text{i-grams}\;\text{in}\;\hat{Y}\;\text{that}\;\text{appear}\;\text{in}\;Y}{\forall \;\text{i-grams}\;\text{in}\;\hat{Y}}$$

The brevity penalty *BP* is defined as:

(9) $$BP=\{\begin{array}{ll} 1, & \text{if}\;c>r\\ \exp(1-r/c), & \text{if}\;c\leq r \end{array}$$

Here *c* is the length of candidate sequence (the number of words in the proposed candidate sequence), and *r* is the length of the reference sequence (the number of words in the reference sequence).

Then the BLEU score is defined as:

(10) $$BLEU=BP\cdot \text{exp}\left(\sum_{u=1}^{N}w_{i}\text{log}\;p_{i}\right)$$

In this equation, *exp* is the natural exponential function; *log* is the natural logarithm function; the weight is *w*_*i*_ = 1*/i*; we set *N* = 2.

For clinical interpretation, our modified BLEU score indicates how well our proposed sub-sequences of causal conditions match the physicians’ results. The 1-gram precision emphasizes individual condition codes matching, while 2-gram precision evaluates the causal relationship between two neighboring condition codes. Physicians can manually check whether the generated causal relationship between any two neighboring condition codes fulfills or contradicts their medical domain knowledge; in addition, a data-driven algorithm can incorporate ACME decision table as medical domain ground truth to assess the validity of two neighboring condition codes.

In Table III, we show an example of different candidate sequences that have perfect 1-gram precision but different 2-gram precision. The reference sequence from underlying cause of death to immediate cause of death is: *I251 (Atherosclerotic heart disease of native coronary artery), I38 (Endocarditis, valve unspecified), I429 (Cardiomyopathy, unspecified) and I469 (Cardiac arrest, cause unspecified)*. The 2-gram precision in the modified BLEU score favors candidate sequences that have more feasible condition codes with pairwise casual relationship.

In addition to our modified BLEU score, we also include three other evaluation criteria: the accuracy for predicting the entire output sequence correctly, the accuracy of predicting individual codes correctly in the output sequence (sequence order not considered), and the accuracy for predicting the underlying cause of death correctly.

## V. Experiments

By using OpenNMT package [32], We have trained the LSTM encoder-decoder models and bi-directional RNN (BRNN) encoder-decoder models with different attention mechanisms. In addition, we also train and evaluate the transformer model with multi-head self attention module on the Michigan dataset. All these experiments are evaluated by BLEU score and three accuracy metrics.

To extend the scope of this work, we explore the feasibility of applying encoder-decoder frameworks on current EHRs data in ICD-10 codes. As the input sequence of the Michigan dataset is coded in ICD-9, we choose to map the input ICD-9 codes into ICD-10 codes using General Equivalence Mappings published by Centers for Medicare & Medicaid Services (CMS).^8^ Specifically, we conduct four experiments on ICD-9 input codes (four combinations with or without validity check, with or without knowledge constraint) and one experiment on ICD-10 input codes without validity check or knowledge constraint.

In addition to OpenNMT, we incorporate the state-of-the-art pretraining model named cross-lingual language model (XLM) [16] on our data set. Lastly, we visualize the attention scores and mapped the relationship between source sequence and output sequence.

### A. Opennmt

OpenNMT serializes the training, validation, and vocabulary data into PyTorch files for preprocessing. As the Michigan dataset has a relatively small sample size comparing with datasets used in similar natural language processing tasks, our models have a small number of parameters but similar architecture as the state-of-the-art models. During training, we use the 2-layer LSTM model, with 500 hidden units in each layer for the LSTM encoder-decoder framework (Luong *et al.* used 4-layer LSTM model with 1000 units [12]). For bidirectional RNN encoder, a 2-layer bidirectional LSTM with 500 and 250 hidden units is implemented. The transformer has six stacking layers, with 2,048 hidden units in feed forward layers and eight heads in multi-head attention layers.

We use one Nvidia GPU Tesla K80 to train and evaluate the models. Typically it takes around one hour to train an LSTM or bidirectional RNN model for 10,000 steps, and about six hours to train a transformer model. Yet it takes less than five minutes to translate all 36,000 testing data using any of these models.

### B. Optional Preprocessing: Validity Check

In search for better prediction performance, we add an extra pre-processing step, the validity check. For training and validation data, we adapt the same algorithm in [22] to remove the pairs of sentences that include “invalid” causal relationship between diagnosis codes in target sentence. In this way we reduce the number of sentences in the training set from 136,753 to 107,711 and those in the validation set from 34,385 to 27,009. We then follow the same pipeline to train and translate with the same encoder-decoder models.

### C. XLM: Pretraining

XLM [16] incorporates masked language modeling (MLM) proposed in BERT (Bidirectional Encoder Representations from Transformers) [14] with the transformer model to improve translation performance. The preprocessing includes tokenizing and applying fastBPE (byte pair encoding) [33] to monolingual and parallel data. MLM is the core strategy in monolingual language model pretraining. Training consists of three major steps: denosing auto-encoder, parallel data training, and online back-translation.

Due to the limited size of our data set, we concatenate all training, validation, and testing data into two corpora for monolingual pre-training. MLM perplexities are used for validation during pre-training. We train the cross-lingual model with parallel validation data and predict on parallel test data. We set the transformer framework with 512 embedding size and 4 attention heads. We vary the encoder-decoder stacking size from 6 layers to 1 layer. The drop out rate was set to 0.1, attention dropout to 0.1, batch size to 32, and sequence length to 128. We used GELU for activation and adam as optimizer.

## VI. Results

### A. Attention Comparison

As shown in Table IV, bi-directional RNN (BRNN) encoder-decoder model with soft attention achieves the highest BLEU score, followed by the transformer model and BRNN with no attention. When comparing different attention mechanisms, LSTM model with soft attention or with general attention has higher BLEU scores than without attention; BRNN models with different attention mechanisms have similar BLEU scores. Comparing LSTM models against BRNN models, LSTM with no attention or with soft attention has lower BLEU scores than BRNN model counterparts, but LSTM model with general attention has very close BLEU score to the BRNN model with general attention. The best model performance has a BLEU score of 17.87, better than the performance of the state-of-the-art in the natural language domain (English-Czech translation task achieving BLEU score 17.7 with same vocabulary size around 10,000) [13].

In addition, we also include the results for the other three evaluation criteria. BRNN model with general attention has the highest accuracy in generating the entire sequence correctly and the highest accuracy in generating individual codes correctly. BRNN model with soft attention has the highest accuracy in predicting the underlying cause of death correctly.

One thing to notice is that all these models with different attention mechanism have very close performance (less than 5% difference). Comparatively, BRNN models with either soft attention or general attention have the best performance among all these frameworks.

### B. Validity Check, Domain Knowledge Constraint and ICD-10 Input Sequence

As shown in Table V, we calculate the average BLEU score and its standard deviation (in parenthesis) for each encoder-decoder framework across five folds. For **Experiment 1** (no validity check in training/ validation data and no knowledge constraint in decoding), the transformer model achieves the highest BLEU score. Comparing **Experiment 1** and **Experiment 2**, validity check, the preprocessing step on training and validation data increases the average BLEU score for LSTM and BRNN models, but decreases the performance of the transformer model. This indicates that validity check has mixed impact on average performance of different models.

It is worth noticing that in **Experiment 3** and **Experiment 4**, the average BLEU score drops significantly for LSTM and BRNN models, while their standard deviation increases significantly. As for the transformer models, knowledge constraint decreases the average BLEU score. Consequently, we show that the encoder-decoder frameworks can learn the causal relationship between diagnosis codes well enough that it is not necessary to learn and incorporate the medical domain knowledge constraint from the ACME decision table during the decoding process.

In addition, it is interesting to compare the results in **Experiment 1** and **Experiment 5**. After mapping the input ICD-9 codes into ICD-10 codes, LSTM, BRNN and transformer models have similar average BLEU scores with those in Experiment 1. These results are significant: 1) the encoder-decoder frameworks are promising and stable in generating the causal sequence of death, no matter whether we have input and output data in the same or different coding systems. 2) When having no access or limited access to the newest EHRs data, we can use data before 2015 to train the models and generate the causal sequence of death.

### C. Attention Visualization: A Case Study

**Fig. 5.**
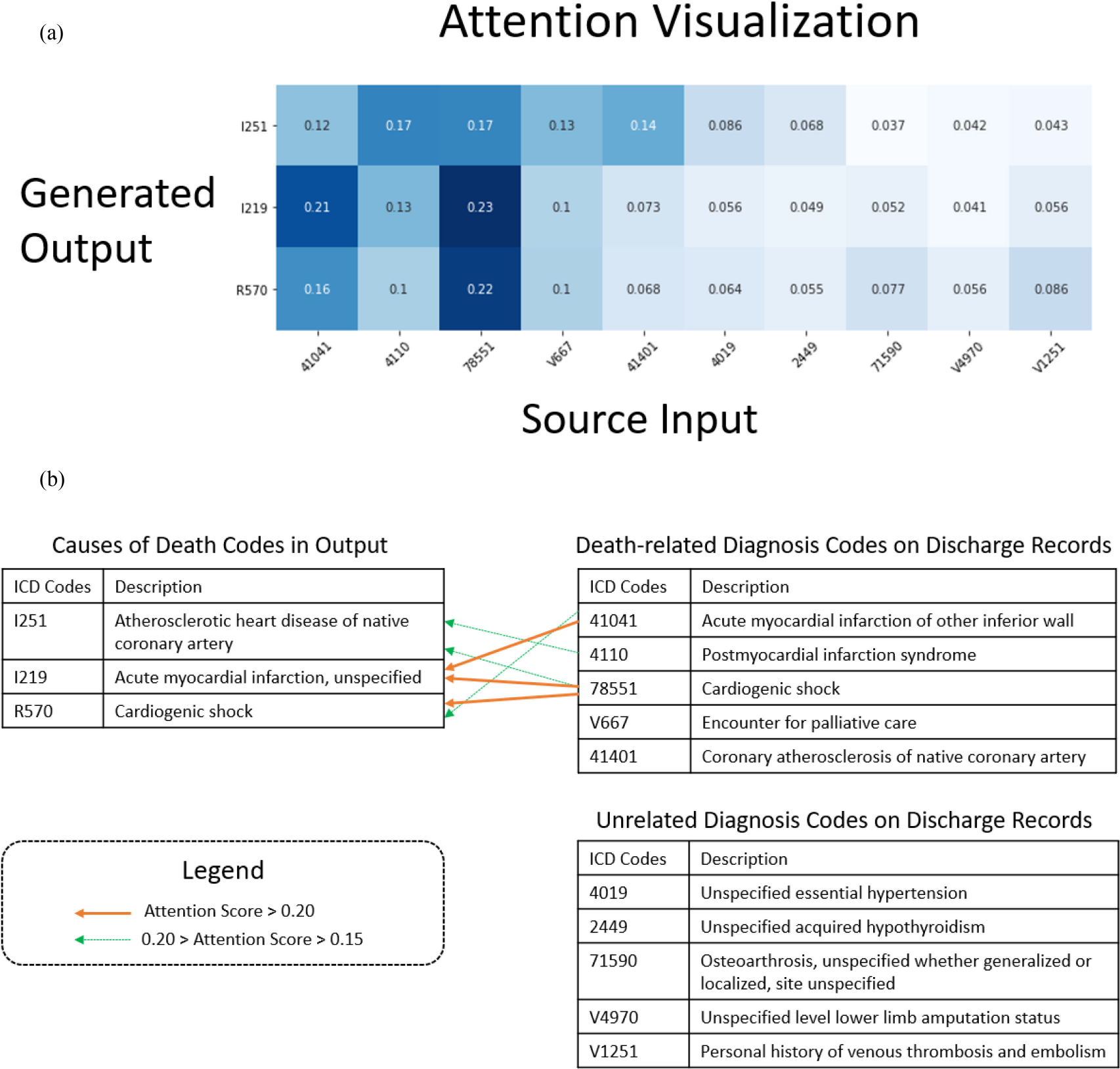
Attention visualization and explanation. In part (a), the attention score matrix is visualized. From top to bottom are the underlying cause of death and immediately causes of death. Darker blue color indicates higher attention scores (the input code is more related with the output code). In part (b), we provide human-readable description to all ICD codes. The identified causes of death for this decedents are cardiovascular diseases.

To better understand the causal relationship between clinical conditions on the discharge records, we visualize the attention scores generated by the bi-directional RNN model. In this case, there are ten diagnosis codes in ICD-9 on the decedent’s discharge record. The generated causal sequence of death is exactly the same as the ground truth (annotated by physicians). We map the attention scores for all diagnosis codes in the input sequence (x-axis) against the causes of death codes in the output sequence (y-axis). As shown in part (A) of Fig. 5, a higher attention score is painted in darker blue, indicating that the input diagnosis code is more related with the code in causal sequence of death. If we empirically set a threshold of 0.1, we can identify five diagnosis codes as death-related conditions (shown in part (B) of Fig. 5). Four of five are severe cardiac conditions, aligned with the causes of death. The other five diagnosis codes are not considered as death-related conditions due to lower attention scores.

The attention scores improve model interpretation by showing the relationship between diagnosis codes and causes of death. Attention visualization also helps the researchers and clinicians identify death-related conditions from available symptoms on discharge records.

### D. XLM

To our surprise, the state-of-the-art algorithm XLM performs much worse than the other encoder-decoder frameworks. All BLEU scores are less than 1 after trying different combinations of hyper-parameters.

The core algorithm behind BERT and XLM, masked language model, may not work on our data set. The idea of masked language modeling is to randomly mask a few words in the sentence (either source or target sentence) during the training stage and then to recover these masked words based on surrounding context. Since, on average, our target sentence has 2.25 words, masking one word can make it extremely difficult to recover. Even worse, over 31% of our target sentences consist of only one word: masking the only word makes it impossible to recover.

## VII. Discussion

According to [13], larger vocabulary size tends to allow models to achieve higher BLEU scores. Their proposed hybrid NMT model achieved 17.7 BLEU score with 10,000 vocabulary size on English-Czech translation task. Our vocabulary size in source set is 7616 and that in target set is 2649. Thus, our results are better than the state-of-the-art results in natural language processing with similar vocabulary size. Even compared with other neural machine translation models [10] [33] with larger vocabulary size (except English-French translation), our results are very similar. A possible extension to the causal relationship is to apply causal inference algorithms [34][35] on causes of death codes and evaluate the average treatment effect.

Wu *et al.* [21] sought to predict the underlying causes of death achieves higher accuracy (75%), but our accuracy in generating individual codes is higher (81%). Blanco’s recent publication on Journal of Biomedical and Health Informatics [23] used similar RNN model to predict the single cause of death codes from verbal autopsy questionnaire data. Their work achieved accuracy of 45.6% and 53.3% for adult and children groups correspondingly, similar to our accuracy for predicting the underlying cause of death. We argue that our models are able to generate most of the individual causes of death codes while covering the underlying cause of death.

Meanwhile, medical domain knowledge as constraint is incorporated when generating output sequence. Even though domain knowledge constraint has a negative impact on the encoder-decoder models, we show that the encoder-decoder frameworks can learn the causal relationship between diagnosis codes from the data. Meanwhile, we demonstrate that validity check can be a critical step in the pipeline which may slightly improve results.

Still, there are a few limitations with this work. First, the medical domain knowledge constraint has a negative impact on generating causal sequence of death. As the causal relationship learned from ACME decision table was only applied on beam search process during decoding, domain knowledge constraint failed to influence the model performance in a positive direction. Alignment or attention mechanism, the core component of encoder-decoder framework, did not use with the domain knowledge constraint. Furthermore, even though that XLM has proven its efficacy in natural language translation, it fails on our task. One potential cause is that the masked language modeling might not work on extremely short sentences (average 2.25 words per sentence).

One potential solution is to apply more recent models and pretrained embeddings. Specifically, Med-BERT [36] is a pretrained embedding of the BERT model on diagnosis codes from structured electronic health records of over 28 million patients. Med-BERT is pretrained on in-hospital length of stay (LOS) prediction tasks and fine tuned with disease prediction tasks. This pretrained embedding of a more advanced model may potentially improve the performance of generating the causal sequence of death.

One unsolvable problem is the one-word target sentence. Rarely do we see sentences consisting just one word in natural language processing tasks; yet 31.77% of our training data, 31.68% of validation data and 31.27% of testing data are one-word target sentences. These samples significantly undermine the perceived efficacy of neural machine translation models.

## VIII. Conclusion

In this paper, we are the first to successfully predict the causal sequence of death using neural machine translation frameworks to support the timely, accurate, and complete death reporting. We also evaluate the model performance using three different accuracy scores, achieving 81.68% accuracy in generating the individual codes in output sequence. Furthermore, we visualize the attention scores to interpret the causal relationship of diagnosis codes from the discharge records. Specifically, we identify the death-related conditions from available symptoms by mapping all diagnosis codes in the input sequence against all causes of death codes in the output sequence. Lastly, we demonstrate a FHIR-based mobile app to retrieve, modify, and upload cause of death data to improve clinical integration.

There are multiple potential directions for future work. 1) The clinical domain knowledge constraint may be implemented to interact with the attention scores in order to constrain the casual relationship during the model learning stage. 2) Using more recent models or pretrained embeddings, such as Med-BERT. 3) As our dataset does not include temporal diagnosis codes, future work may find data with time-stamped information. 4) Our dataset was collected before 2017 and thus has no COVID-related death. Future collaboration will include discharge records and death certificate records collected during and after the pandemic. In this way we can test our approach to identify COVID-related severe symptoms and causes of death.

## Supplementary Material

supp1-3163013

## Figures and Tables

**TABLE I:** Summary of Challenges in Generating the Causal Sequence of Death and Proposed Solutions

Challenge	Solution
Different coding versions	Machine translation between input and output sequences
Domain knowledge conflict	Incorporate medical domain knowledge as constraint
Data interoperability	FHIR compatible platform

**TABLE II:** An Example of 1-Gram Precision and 2-Gram Precision in BLEU Score

Grams	From Candidate $\hat{Y}$	Appear in *Y*	Precision
1-gram	(R909), (J189), (J969)	(R909), (J189)	2/3
2-gram	(R909, J189), (J189, J969)	(R909, J189)	1/2

**TABLE III:** Our Modified BLEU Score for Different Candidate Sequences

	Sequence	BLEU
Reference	*I*251 → *I*38 → *I*429 → *I*469	
Candidate 1	*I*429 → *I*38 → *I*469 → *I*251	0.0
Candidate 2	*I*38 → *I*429 → *I*251 → *I*469	57.7
Candidate 3	*I*429 → *I*469 → *I*251 → *I*38	81.6
Candidate 4	*I*38 → *I*429 → *I*469 → *I*251	81.6
Candidate 5	*I*251 → *I*38 → *I*429 → *I*469	100.0

**TABLE IV:** Average BLEU and Accuracy Scores and Standard Deviation in Parentheses Across Five Folds

Model	Attention	BLEU	Entire Sequence Accuracy	Individual Codes Accuracy	Underlying COD Accuracy

LSTM	No Attention	17.09 (0.75)	16.55 (0.69)	81.30 (0.39)	54.97 (0.63)
	Soft Attention	17.55 (0.66)	16.87 (0.68)	81.59 (0.41)	55.54 (0.57)
	General Attention	17.62 (1.03)	16.76 (0.96)	81.41 (0.40)	55.15 (0.93)

BRNN	No Attention	17.76 (0.60)	16.66 (0.84)	80.89 (0.39)	55.29 (0.60)
	Soft Attention	**17.87** (0.74)	16.43 (0.57)	80.77 (1.04)	**55.64** (1.49)
	General Attention	17.61 (0.74)	**16.91** (0.69)	**81.68** (0.28)	55.49 (0.52)

Transformer	Self Attention	17.77 (0.55)	16.47 (0.59)	79.64 (0.50)	54.91 (0.67)

**TABLE V:** Average BLEU Scores and Standard Deviation in Parentheses for Five Experiments

Experiment	Input Data	Validity Check	Knowledge Constraint	LSTM General Attention	BRNN General Attention	Transformer

1	ICD-9	Not checked	Non-constrained	17.62 (1.03)	17.61 (0.74)	**17.77** (0.55)
2		Checked	Non-constrained	17.85 (1.18)	**18.26** (1.10)	15.32 (0.37)
3		Not checked	Constrained	12.61 (6.33)	12.46 (6.26)	14.76 (0.51)
4		Checked	Constrained	12.95 (6.50)	13.16 (6.61)	14.99 (0.42)

5	ICD-10	Not checked	Non-constrained	17.86 (0.50)	17.89 (1.06)	16.31 (0.61)

## Footnotes

1 [Online]. Available: https://www.who.int/news-room/fact-sheets/detail/the-top-10-causes-of-death

2 [Online]. Available: https://covid19.who.int/

3 [Online]. Available: https://www.cdc.gov/nchs/icd/icd10cm.htm

4 [Online]. Available: https://www.cdc.gov/nchs/icd/icd10cm_pcs_faq.htm

5 [Online]. Available: https://www.cdc.gov/nchs/data/icd/icd9cm_guidelines_2011.pdf

6 [Online]. Available: https://www.cdc.gov/nchs/data_access/vitalstatsonline.htm#Mortalit_Multiple

7 Adapted from https://towardsdatascience.com/transformers-141e32e69591

8 [Online]. Available: https://www.nber.org/research/data/icd-9-cm-and-icd-10-cm-and-icd-10-pcs-crosswalk-or-general-equivalence-mappings
