# Supplementary material for: Proposing Causal Sequence of Death by Neural Machine Translation in Public Health Informatics
Source: IEEE J Biomed Health Inform. Author manuscript; Available in PMC 2022 Sep 7. (PMC9452006; doi:10.1109/JBHI.2022.3163013)
Supplement: supp1-3163013 [file NIHMS1798541-supplement-supp1-3163013.pdf]

Supplemental Document for Public Health Informatics: Proposing Causal Sequence of Death Using Neural Machine Translation

Yuanda Zhu *, *Student Member, IEEE,* Ying Sha *, *Student Member, IEEE,* Hang Wu *, *Student Member, IEEE,* Mai Li, *Student Member, IEEE,* Ryan A. Hoffman, *Student Member, IEEE,* May D. Wang, *Fellow, IEEE*

I. Ten-fold Cross Validation

We also did ten-fold cross validation (train/validation/test split is 8:1:1) to generate BLEU scores as well as three accuracy scores for different encoder-decoder models with different attention mechanisms. As shown in **Table I**, LSTM with soft attention and BRNN with general attention achieved the best results.

We also did statistical analysis using two-way ANOVA (ANalysis Of VAriance) to compare the BLEU scores between five-fold cross validation and ten-fold cross validation. The two independent variables are number of folds in cross validation and model + attention. The null hypothesis is that the mean BLEU scores in different groups are the same. Thus, the alternative hypothesis is that one group mean is different from other groups. The ANOVA results indicated that five-fold and ten-fold cross validation did not have significantly different results for these models/attentions ($p > 0.05$). **Figure 1** shows the box plot of results in two cross validation scenarios.

II. FHIR Interface

We have implemented a Android mobile application to demonstrate the usefulness and applicability of this work. This application supports causal chain prediction, patient search, patient information display, determining causes of death, and data review/submit. This application allows a physician to add death-related information when filling out the "Pronouncing Death" screen and the submitted data bundle is compatible with FHIR compatible servers. When generating causal chain of death for clinical decision support, the application will automatically retrieve medical condition codes from the FHIR compatible server, and query the python pre-trained neural machine translation model for generating the casual chain of death. **Figure. 2** shows a screenshot of the FHIR Android application when displaying the causal chain of death. The ICD-10 codes are mapped into human-readable short descriptions. From top to bottom, we show the ordered causes of death (from underlying cause of death to immediate causes of death). "N/A" indicates null value for the cause of death.

The work was supported by the U.S. Department of Health and Human Services Centers for Disease Control and Prevention under Award HHSD2002015F62550B, the NIH National Center for Advancing Translational Sciences under Award UL1TR000454, and the National Science Foundation Award NSF1651360.

Y. Zhu is with School of Electrical and Computer Engineering, Georgia Institute of Technology, Atlanta, GA 30332 USA (e-mail: yzhu94@gatech.edu).

Y. Sha is with School of Biology, Georgia Institute of Technology, Atlanta, GA 30332 USA (e-mail: ysha8@gatech.edu).

H. Wu is with Department of Biomedical Engineering, Georgia Institute of Technology, Atlanta, GA 30332 USA (e-mail: hangwu@gatech.edu)

M. Li is with Department of Electronic Science and Technology, University of Science and Technology of China, Hefei, Anhui Province, China (e-mail: lm333@mail.ustc.edu.cn).

R. A. Hoffman and M. D. Wang are with Department of Biomedical Engineering, Georgia Institute of Technology and Emory University, Atlanta, GA 30332 USA (phone: 404-385-2954; e-mail: rhoffman12@gatech.edu, maywang@gatech.edu)

*The first three authors contributed equally to this work.

The use of mobile apps for delivering public health informatics creates the opportunity for real-time point-of-care feedback. In the immediate term, clinicians completing mortality reporting data can be provided with decision support capability to improve the accuracy and completeness of the causes of death reported. In the future, such infrastructure may even be able to provide predicted causes of death for still-living patients, enabling data-driven and personalized medical care.

The FHIR application is available on GitHub [1]. The video demo is available on YouTube [2].

[1] https://github.com/UnitedHolmes/-CDC-FHIR-Android-App
[2] https://youtu.be/9Bubwd2OzaA

TABLE I
AVERAGE BLEU AND ACCURACY SCORES AND STANDARD DEVIATION IN PARENTHESES ACROSS TEN FOLDS.

Model	Attention	BLEU	Entire Sequence Accuracy	Individual Codes Accuracy	Underlying COD Accuracy
LSTM	No Attention	18.18 (0.77)	16.90 (0.96)	80.93 (0.90)	56.35 (0.89)
	Soft Attention	**18.34** (0.89)	16.53 (1.14)	80.39 (1.27)	55.00 (1.03)
	General Attention	17.82 (0.68)	17.07 (0.79)	**81.70** (0.45)	56.22 (0.75)
BRNN	No Attention	17.37 (0.91)	16.76 (0.88)	81.33 (0.49)	55.92 (1.24)
	Soft Attention	17.95 (1.15)	16.43 (0.91)	80.24 (0.90)	**56.36** (0.63)
	General Attention	17.93 (0.81)	**17.22** (0.74)	81.61 (0.54)	56.17 (1.02)
Transformer	Self Attention	16.16 (0.92)	15.80 (0.90)	79.45 (0.44)	52.96 (1.22)

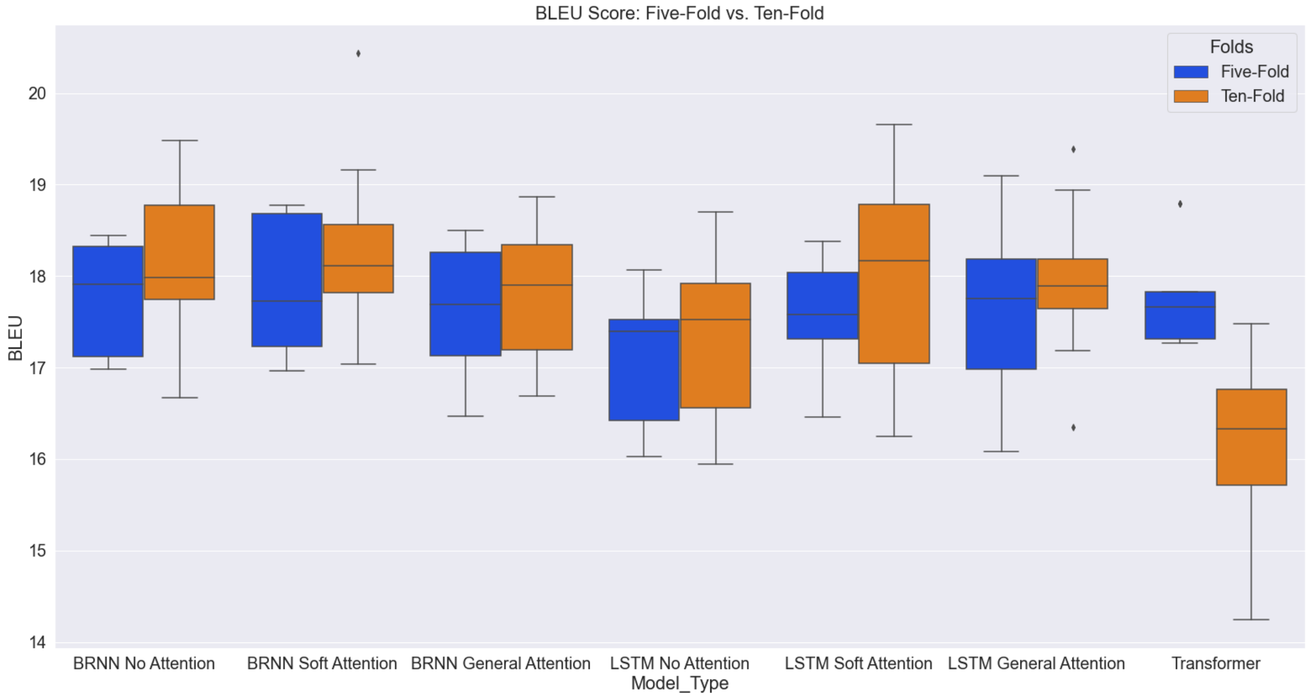

Fig. 1. Box plot of BLEU scores for different model and attention types with five-fold and ten-fold cross validation.

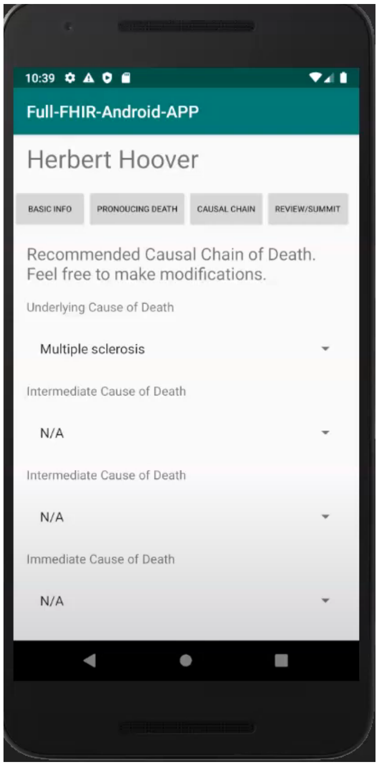

Fig. 2. Screenshot of causal chain prediction display in the Android application. From top to bottom, the diagnosis codes correspond to the order of the output sentence. The ICD-10 codes are mapped into human-readable short descriptions.
